# Crystal structure of 13-(2-meth­oxy­phenyl)-3,4-di­hydro-2*H*-indazolo[1,2-*b*]phthalazine-1,6,11(13*H*)-trione

**DOI:** 10.1107/S2056989015013894

**Published:** 2015-07-25

**Authors:** Abdelmalek Bouraiou, Sofiane Bouacida, Hocine Merazig, Aissa Chibani, Zouhair Bouaziz

**Affiliations:** a Unité de recherche de Chimie de l’Environnement et Moléculaire Structurale, CHEMS, Université des frères Mentouri, Constantine 25000, Algeria; b Département Sciences de la matière, Université Oum El Bouaghi, 04000, Algeria; c Laboratoire de Chimie Organique, EA 4446 Biomolécules, Cancer et Chimiorésistances (B2C), Université Claude Bernard Lyon 1, Faculté de Pharmacie–ISPB, Lyon Cedex 08, France

**Keywords:** crystal structure, phthalazine, indazole, C—H⋯O hydrogen bonds, C—H⋯π inter­actions, π–π inter­actions

## Abstract

In the title compound, C_22_H_18_N_2_O_4_, the three fused rings of the pyrazolo­phthalazine moiety are coplanar (r.m.s. deviation = 0.027 Å). The cyclo­hexene ring fused to the pyrazolidine ring, so forming the indazolophthalazine unit, has a half-chair conformation. The benzene ring is almost normal to the mean plane of the pyrazolo­phthalazine moiety, with a dihedral angle of 87.21 (6)° between their planes. In the crystal, mol­ecules are linked by pairs of C—H⋯O hydrogen bonds forming inversion dimers. The dimers are linked *via* C—H⋯π inter­actions, forming slabs parallel to (100). Between the slabs there are weak π–π inter­actions [shortest inter-centroid distance = 3.6664 (9) Å], leading to the formation of a three-dimensional structure.

## Related literature   

For the synthesis of phthalazine derivatives, see: Hasaninejed *et al.*, (2012 ▸); Keshipour *et al.*, (2012 ▸). For applications of this class of compounds, see: Soliman *et al.* (1981 ▸); Nomoto *et al.* (1990 ▸); Abd El-Wahab *et al.* (2013 ▸); Cashman & Ghirmai (2009 ▸); Hall *et al.* (1992 ▸, 2001 ▸). For the synthesis of the title compound, see: Khurana & Magoo (2009 ▸). For similar condensation reactions as used here, see: Atar *et al.* (2015 ▸). For the Cambridge Structural Database, see: Groom & Allen (2014 ▸).
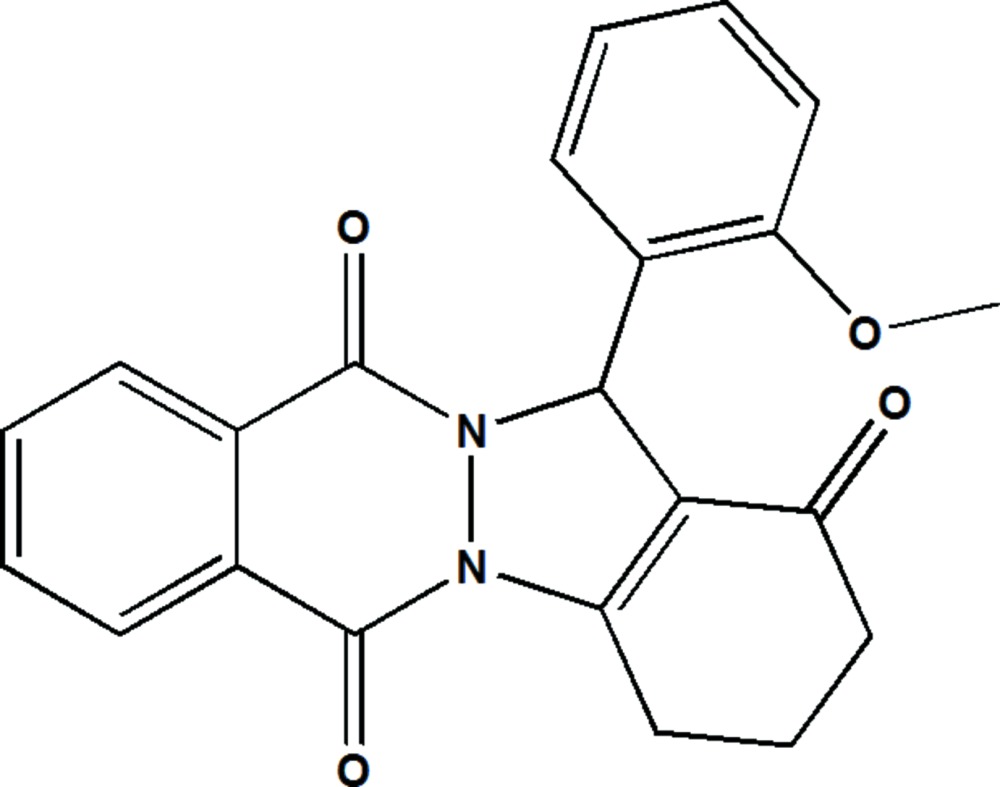


## Experimental   

### Crystal data  

C_22_H_18_N_2_O_4_
*M* *_r_* = 374.38
Monoclinic, 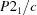

*a* = 8.5839 (2) Å
*b* = 11.8474 (2) Å
*c* = 17.5317 (4) Å
β = 102.199 (1)°
*V* = 1742.66 (6) Å^3^
*Z* = 4
Mo *K*α radiation
μ = 0.10 mm^−1^
*T* = 295 K
0.15 × 0.11 × 0.08 mm

### Data collection  

Bruker APEXII diffractometer
Absorption correction: multi-scan (*SADABS*; Bruker, 2011 ▸) *T*_min_ = 0.983, *T*_max_ = 0.991
17655 measured reflections
5142 independent reflections
3865 reflections with *I* > 2σ(*I*)
*R*_int_ = 0.02

### Refinement  

*R*[*F* ^2^ > 2σ(*F* ^2^)] = 0.056
*wR*(*F* ^2^) = 0.163
*S* = 1.03
5142 reflections
254 parameters
H-atom parameters constrained
Δρ_max_ = 0.56 e Å^−3^
Δρ_min_ = −0.41 e Å^−3^

### 

Data collection: *APEX2* (Bruker, 2011 ▸); cell refinement: *SAINT* (Bruker, 2011 ▸); data reduction: *SAINT*; program(s) used to solve structure: *SIR2002* (Burla *et al.*, 2005 ▸); program(s) used to refine structure: *SHELXL97* (Sheldrick, 2008 ▸); molecular graphics: *ORTEP-3 for Windows* (Farrugia, 2012 ▸) and *DIAMOND* (Brandenburg & Berndt, 2001 ▸); software used to prepare material for publication: *WinGX* (Farrugia, 2012 ▸).

## Supplementary Material

Crystal structure: contains datablock(s) I. DOI: 10.1107/S2056989015013894/su5175sup1.cif

Structure factors: contains datablock(s) I. DOI: 10.1107/S2056989015013894/su5175Isup2.hkl

Supporting information file. DOI: 10.1107/S2056989015013894/su5175Isup3.cml

**Fig. 1.**
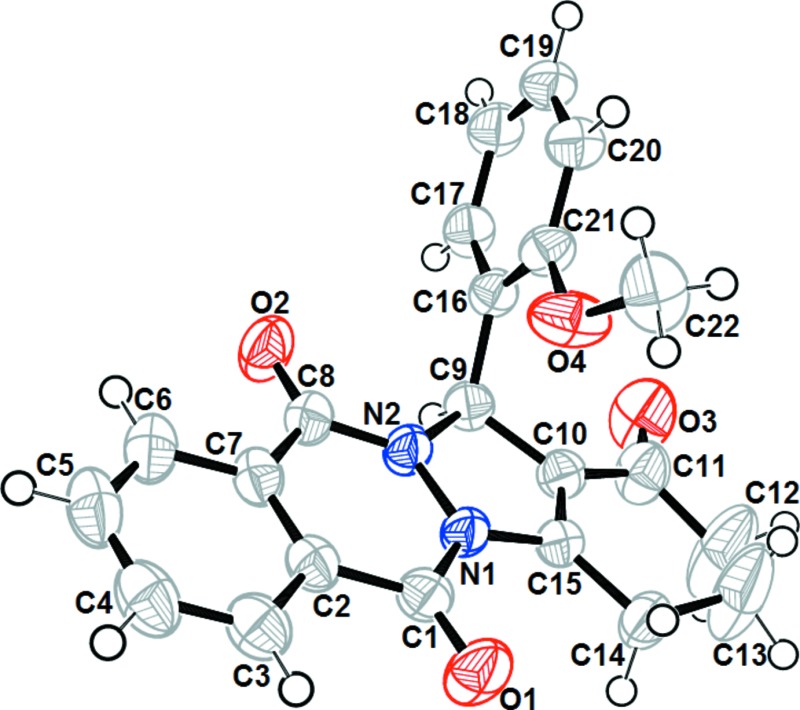
The molecule structure of the title compound, showing the atom labelling. Displacement are drawn at the 50% probability level.

**Fig. 2.**
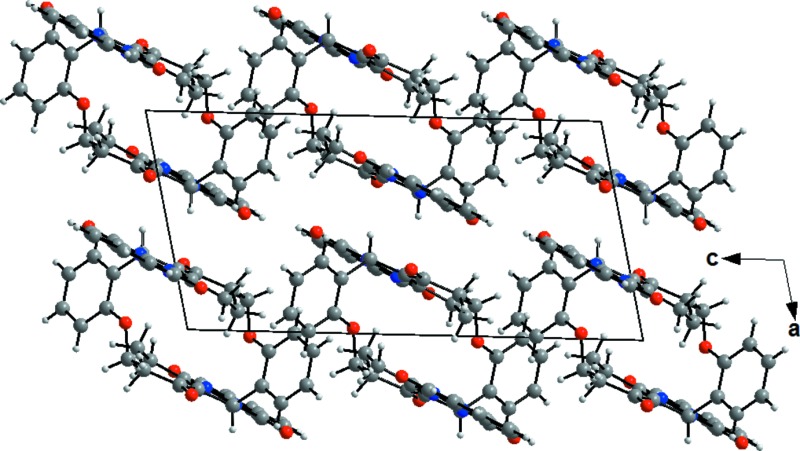
A view along the b axis of the crystal packing of the title compound.

. DOI: 10.1107/S2056989015013894/su5175fig1.tif
The mol­ecule structure of the title compound, showing the atom labelling. Displacement are drawn at the 50% probability level.

b . DOI: 10.1107/S2056989015013894/su5175fig2.tif
A view along the *b* axis of the crystal packing of the title compound.

CCDC reference: 1414255

Additional supporting information:  crystallographic information; 3D view; checkCIF report

## Figures and Tables

**Table 1:** Hydrogen-bond geometry (, ) *Cg*3 is the centroid of ring C2-C7.

*D*H*A*	*D*H	H*A*	*D* *A*	*D*H*A*
C22H22*B*O1^i^	0.96	2.43	3.379(2)	168
C20H20*Cg*3^ii^	0.93	2.90	3.726(2)	149

Symmetry codes: (i) 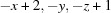; (ii) 
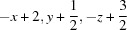
.

## supplementary crystallographic information

### S1. Comment

Nitro­gen heterocycles containing a phthalazine moiety have attracted significant synthetic inter­est because they show biological and pharmacological activities such as anti­convulsant (Soliman *et al.*, 1981), cardiotonic (Nomoto *et al.*, 1990), and anti­microbial (Abd El-Wahab *et al.*, 2013). In addition, phthalazines have been reported to act as potential inhibitors of serotonin re-uptake (Cashman & Ghirmai, 2009) and as effective anti-proliferative agents against different human and murine tumor cells (Hall *et al.*, 1992;2001). During the last two decades there is a growing inter­est in the synthesis of several phthalazines as promising drug candidates for the treatment of cancer (Hasaninejed *et al.*, 2012; Keshipour *et al.*, 2012). Herein we report on the synthesis and crystal structure of the title compound, synthesized by condensation of phthalhydrazide, 2-meth­oxy­benzaldehyde and 1,3-cyclo­hexadione (Atar *et al.*, 2015).

The molecule structure of the title compound is illustrated in Fig. 1. It consists of an indazolone moiety bearing a meth­oxy­penyl group and attached to a phthalazine. The phthalazine ring is quasi-planar with a maximum deviation of 0.0203 (17) Å for atom C3, and forms a dihedral angle of 86.76 (4) ° with the benzene ring. All bond distances and angles are within the ranges of accepted values, CSD, (Groom & Allen, 2014).

In the crystal, molecules are linked by pairs of C—H···O hydrogen bonds forming inversion dimers (Table 1). The dimers are linked *via* C—H···π inter­actions forming slabs parallel to (100); Table 1 and Fig. 2. Between the slabs there are weak π–π inter­actions [shortest inter-centroid distance = 3.6664 (9) ° for Cg1···Cg3^i^; Cg1 and Cg3 and the centroids of rings N1/N2/C9/C10/C15 and C2—C7, respectively; symmetry code: -x+1, -y, -z+1], leading to the formation of a three-dimensional structure.

### S2. Synthesis and crystallization

Phthalhydrazide (1.0 mmol), 2-meth­oxy­benzaldehyde (1.2 mmol), 1,3-cyclo­hexane­dione (1.0 mmol), H_2_SO_4_ (0.15 mmol), and 10 ml H_2_O-EtOH were mixed under reflux following a published procedure (Khurana & Magoo, 2009). The precipitate formed was collected by filtration, and dried. The crude product was washed well with hot ethanol. The solid obtained, was recrystallized in CHCl_3_ giving colourless crystals of the title compound on slow evaporation of the solvent.

### S3. Refinement

Crystal data, data collection and structure refinement details are summarized in Table 2. The H atoms were localized in difference Fourier maps but introduced in calculated positions and treated as riding atoms: C—H = 0.93 - 0.98 Å with *U*_iso_(H) = 1.5 *U*_eq_(C) for methyl H atoms and 1.2U_eq_(C) for other H atoms.

### Crystal data

**Table:**

C_22_H_18_N_2_O_4_	*F*(000) = 784
*M**_r_* = 374.38	*D*_x_ = 1.427 Mg m^−^^3^
Monoclinic, *P*2_1_/*c*	Mo *K*α radiation, λ = 0.71073 Å
Hall symbol: -P 2ybc	Cell parameters from 7478 reflections
*a* = 8.5839 (2) Å	θ = 3.0–30.1°
*b* = 11.8474 (2) Å	µ = 0.10 mm^−^^1^
*c* = 17.5317 (4) Å	*T* = 295 K
β = 102.199 (1)°	Prism, colourless
*V* = 1742.66 (6) Å^3^	0.15 × 0.11 × 0.08 mm
*Z* = 4	

### Data collection

**Table:**

Bruker APEXII diffractometer	5142 independent reflections
Radiation source: Enraf Nonius FR590	3865 reflections with *I* > 2σ(*I*)
Graphite monochromator	*R*_int_ = 0.02
CCD rotation images, thick slices scans	θ_max_ = 30.2°, θ_min_ = 3.4°
Absorption correction: multi-scan (*SADABS*; Bruker, 2011)	*h* = −11→12
*T*_min_ = 0.983, *T*_max_ = 0.991	*k* = −15→16
17655 measured reflections	*l* = −24→24

### Refinement

**Table:**

Refinement on *F*^2^	Primary atom site location: structure-invariant direct methods
Least-squares matrix: full	Secondary atom site location: difference Fourier map
*R*[*F*^2^ > 2σ(*F*^2^)] = 0.056	Hydrogen site location: inferred from neighbouring sites
*wR*(*F*^2^) = 0.163	H-atom parameters constrained
*S* = 1.03	*w* = 1/[σ^2^(*F*_o_^2^) + (0.0781*P*)^2^ + 0.6856*P*] where *P* = (*F*_o_^2^ + 2*F*_c_^2^)/3
5142 reflections	(Δ/σ)_max_ < 0.001
254 parameters	Δρ_max_ = 0.56 e Å^−^^3^
0 restraints	Δρ_min_ = −0.41 e Å^−^^3^

### Special details

**Table:**

Geometry. All e.s.d.'s (except the e.s.d. in the dihedral angle between two l.s. planes) are estimated using the full covariance matrix. The cell e.s.d.'s are taken into account individually in the estimation of e.s.d.'s in distances, angles and torsion angles; correlations between e.s.d.'s in cell parameters are only used when they are defined by crystal symmetry. An approximate (isotropic) treatment of cell e.s.d.'s is used for estimating e.s.d.'s involving l.s. planes.
Refinement. Refinement of *F*^2^ against ALL reflections. The weighted *R*-factor *wR* and goodness of fit *S* are based on *F*^2^, conventional *R*-factors *R* are based on *F*, with *F* set to zero for negative *F*^2^. The threshold expression of *F*^2^ > σ(*F*^2^) is used only for calculating *R*-factors(gt) *etc*. and is not relevant to the choice of reflections for refinement. *R*-factors based on *F*^2^ are statistically about twice as large as those based on *F*, and *R*- factors based on ALL data will be even larger.

### Fractional atomic coordinates and isotropic or equivalent isotropic displacement parameters (Å^2^)

**Table:**

	*x*	*y*	*z*	*U*_iso_*/*U*_eq_	
C1	0.74930 (19)	0.02215 (12)	0.50539 (9)	0.0359 (3)	
C2	0.68942 (17)	−0.04887 (12)	0.56184 (8)	0.0337 (3)	
C3	0.6996 (2)	−0.16641 (14)	0.55560 (10)	0.0430 (4)	
H3	0.7435	−0.198	0.5163	0.052*	
C4	0.6449 (2)	−0.23529 (15)	0.60752 (12)	0.0490 (4)	
H4	0.6524	−0.3133	0.6034	0.059*	
C5	0.5787 (2)	−0.18879 (16)	0.66597 (11)	0.0489 (4)	
H5	0.5424	−0.2357	0.701	0.059*	
C6	0.5665 (2)	−0.07285 (16)	0.67227 (10)	0.0447 (4)	
H6	0.5217	−0.0421	0.7115	0.054*	
C7	0.62115 (17)	−0.00193 (13)	0.61993 (9)	0.0341 (3)	
C8	0.60256 (17)	0.12191 (13)	0.62625 (9)	0.0352 (3)	
C9	0.65657 (17)	0.30813 (12)	0.56789 (8)	0.0320 (3)	
H9	0.5459	0.3345	0.5569	0.038*	
C10	0.72602 (18)	0.32444 (12)	0.49673 (8)	0.0333 (3)	
C11	0.7440 (2)	0.43077 (15)	0.45874 (11)	0.0485 (4)	
C12	0.8116 (5)	0.4197 (2)	0.38642 (18)	0.0957 (11)	
H12A	0.7232	0.4177	0.3416	0.115*	
H12B	0.8726	0.4873	0.3818	0.115*	
C13	0.9104 (4)	0.3247 (2)	0.38186 (19)	0.0949 (11)	
H13A	1.0142	0.3395	0.4148	0.114*	
H13B	0.9251	0.3193	0.3286	0.114*	
C14	0.8543 (2)	0.21095 (15)	0.40457 (10)	0.0430 (4)	
H14A	0.782	0.1775	0.3603	0.052*	
H14B	0.9448	0.1608	0.4201	0.052*	
C15	0.77157 (17)	0.22582 (12)	0.47050 (8)	0.0320 (3)	
C16	0.74875 (17)	0.36667 (12)	0.64012 (8)	0.0315 (3)	
C17	0.67423 (19)	0.44961 (13)	0.67530 (9)	0.0382 (3)	
H17	0.5667	0.4645	0.6562	0.046*	
C18	0.7569 (2)	0.51077 (14)	0.73840 (10)	0.0435 (4)	
H18	0.7055	0.5664	0.7612	0.052*	
C19	0.9162 (2)	0.48835 (14)	0.76704 (9)	0.0422 (4)	
H19	0.9725	0.5296	0.8091	0.051*	
C20	0.9930 (2)	0.40511 (14)	0.73373 (9)	0.0402 (3)	
H20	1.1001	0.3898	0.7538	0.048*	
C21	0.90965 (19)	0.34405 (13)	0.67001 (9)	0.0350 (3)	
C22	1.1419 (2)	0.25375 (17)	0.64403 (14)	0.0562 (5)	
H22A	1.1802	0.3224	0.6253	0.084*	
H22B	1.17	0.1909	0.6151	0.084*	
H22C	1.1893	0.2443	0.6984	0.084*	
N1	0.73260 (15)	0.13669 (10)	0.51526 (7)	0.0322 (3)	
N2	0.65943 (15)	0.18347 (10)	0.57313 (7)	0.0331 (3)	
O1	0.80755 (19)	−0.01451 (11)	0.45288 (8)	0.0575 (4)	
O2	0.53946 (16)	0.16813 (11)	0.67456 (8)	0.0511 (3)	
O3	0.7009 (2)	0.52090 (11)	0.48121 (10)	0.0660 (4)	
O4	0.97523 (15)	0.25908 (12)	0.63425 (8)	0.0533 (4)	

### Atomic displacement parameters (Å^2^)

**Table:**

	*U*^11^	*U*^22^	*U*^33^	*U*^12^	*U*^13^	*U*^23^
C1	0.0434 (8)	0.0281 (7)	0.0371 (7)	−0.0015 (6)	0.0108 (6)	−0.0034 (5)
C2	0.0333 (7)	0.0295 (7)	0.0368 (7)	−0.0023 (5)	0.0038 (5)	0.0018 (5)
C3	0.0455 (9)	0.0316 (8)	0.0503 (9)	0.0000 (6)	0.0063 (7)	0.0012 (7)
C4	0.0474 (9)	0.0334 (8)	0.0614 (11)	−0.0041 (7)	0.0008 (8)	0.0097 (7)
C5	0.0398 (8)	0.0467 (10)	0.0576 (10)	−0.0079 (7)	0.0041 (7)	0.0190 (8)
C6	0.0393 (8)	0.0485 (10)	0.0479 (9)	−0.0041 (7)	0.0129 (7)	0.0096 (7)
C7	0.0293 (6)	0.0348 (7)	0.0378 (7)	−0.0027 (5)	0.0058 (5)	0.0040 (6)
C8	0.0335 (7)	0.0375 (8)	0.0365 (7)	−0.0027 (6)	0.0118 (5)	−0.0010 (6)
C9	0.0334 (7)	0.0283 (7)	0.0354 (7)	0.0020 (5)	0.0094 (5)	−0.0025 (5)
C10	0.0368 (7)	0.0303 (7)	0.0328 (6)	−0.0014 (6)	0.0075 (5)	0.0002 (5)
C11	0.0612 (11)	0.0334 (8)	0.0553 (10)	0.0031 (7)	0.0225 (8)	0.0080 (7)
C12	0.162 (3)	0.0495 (13)	0.105 (2)	0.0176 (16)	0.095 (2)	0.0275 (13)
C13	0.151 (3)	0.0484 (12)	0.118 (2)	0.0047 (15)	0.104 (2)	0.0130 (13)
C14	0.0528 (9)	0.0401 (8)	0.0422 (8)	−0.0032 (7)	0.0242 (7)	−0.0027 (7)
C15	0.0349 (7)	0.0310 (7)	0.0309 (6)	−0.0036 (5)	0.0087 (5)	−0.0010 (5)
C16	0.0368 (7)	0.0270 (6)	0.0323 (6)	0.0008 (5)	0.0107 (5)	−0.0015 (5)
C17	0.0401 (8)	0.0325 (7)	0.0451 (8)	0.0028 (6)	0.0161 (6)	−0.0045 (6)
C18	0.0539 (10)	0.0345 (8)	0.0467 (8)	0.0000 (7)	0.0210 (7)	−0.0113 (7)
C19	0.0536 (9)	0.0381 (8)	0.0366 (7)	−0.0072 (7)	0.0132 (7)	−0.0092 (6)
C20	0.0416 (8)	0.0416 (8)	0.0366 (7)	0.0011 (7)	0.0065 (6)	−0.0040 (6)
C21	0.0408 (8)	0.0312 (7)	0.0342 (7)	0.0049 (6)	0.0104 (6)	−0.0027 (5)
C22	0.0522 (10)	0.0382 (9)	0.0834 (14)	0.0091 (8)	0.0259 (10)	−0.0052 (9)
N1	0.0396 (6)	0.0275 (6)	0.0326 (6)	−0.0021 (5)	0.0145 (5)	−0.0036 (5)
N2	0.0398 (6)	0.0286 (6)	0.0346 (6)	−0.0010 (5)	0.0161 (5)	−0.0038 (5)
O1	0.0913 (10)	0.0349 (6)	0.0579 (8)	0.0002 (6)	0.0419 (7)	−0.0080 (6)
O2	0.0617 (8)	0.0472 (7)	0.0543 (7)	−0.0017 (6)	0.0349 (6)	−0.0038 (6)
O3	0.0920 (11)	0.0336 (7)	0.0801 (10)	0.0118 (7)	0.0353 (9)	0.0094 (6)
O4	0.0429 (6)	0.0565 (8)	0.0566 (7)	0.0165 (6)	0.0016 (5)	−0.0248 (6)

### Geometric parameters (Å, º)

**Table:**

C1—O1	1.2168 (19)	C12—H12A	0.97
C1—N1	1.3794 (19)	C12—H12B	0.97
C1—C2	1.472 (2)	C13—C14	1.512 (3)
C2—C7	1.394 (2)	C13—H13A	0.97
C2—C3	1.401 (2)	C13—H13B	0.97
C3—C4	1.377 (2)	C14—C15	1.490 (2)
C3—H3	0.93	C14—H14A	0.97
C4—C5	1.387 (3)	C14—H14B	0.97
C4—H4	0.93	C15—N1	1.3977 (18)
C5—C6	1.384 (3)	C16—C17	1.386 (2)
C5—H5	0.93	C16—C21	1.396 (2)
C6—C7	1.396 (2)	C17—C18	1.386 (2)
C6—H6	0.93	C17—H17	0.93
C7—C8	1.483 (2)	C18—C19	1.379 (3)
C8—O2	1.2266 (18)	C18—H18	0.93
C8—N2	1.3526 (19)	C19—C20	1.382 (2)
C9—N2	1.4797 (19)	C19—H19	0.93
C9—C10	1.504 (2)	C20—C21	1.395 (2)
C9—C16	1.512 (2)	C20—H20	0.93
C9—H9	0.98	C21—O4	1.3684 (18)
C10—C15	1.344 (2)	C22—O4	1.406 (2)
C10—C11	1.448 (2)	C22—H22A	0.96
C11—O3	1.222 (2)	C22—H22B	0.96
C11—C12	1.507 (3)	C22—H22C	0.96
C12—C13	1.422 (4)	N1—N2	1.4143 (16)
			
O1—C1—N1	121.07 (14)	C14—C13—H13A	107.9
O1—C1—C2	124.22 (14)	C12—C13—H13B	107.9
N1—C1—C2	114.70 (13)	C14—C13—H13B	107.9
C7—C2—C3	119.78 (15)	H13A—C13—H13B	107.2
C7—C2—C1	121.59 (14)	C15—C14—C13	109.23 (15)
C3—C2—C1	118.63 (14)	C15—C14—H14A	109.8
C4—C3—C2	120.11 (17)	C13—C14—H14A	109.8
C4—C3—H3	119.9	C15—C14—H14B	109.8
C2—C3—H3	119.9	C13—C14—H14B	109.8
C3—C4—C5	120.23 (17)	H14A—C14—H14B	108.3
C3—C4—H4	119.9	C10—C15—N1	110.08 (12)
C5—C4—H4	119.9	C10—C15—C14	126.12 (14)
C6—C5—C4	120.21 (16)	N1—C15—C14	123.79 (13)
C6—C5—H5	119.9	C17—C16—C21	118.82 (14)
C4—C5—H5	119.9	C17—C16—C9	119.25 (13)
C5—C6—C7	120.20 (17)	C21—C16—C9	121.84 (12)
C5—C6—H6	119.9	C18—C17—C16	121.27 (15)
C7—C6—H6	119.9	C18—C17—H17	119.4
C2—C7—C6	119.45 (15)	C16—C17—H17	119.4
C2—C7—C8	121.20 (13)	C19—C18—C17	119.40 (14)
C6—C7—C8	119.34 (14)	C19—C18—H18	120.3
O2—C8—N2	120.73 (15)	C17—C18—H18	120.3
O2—C8—C7	124.31 (14)	C18—C19—C20	120.55 (15)
N2—C8—C7	114.96 (13)	C18—C19—H19	119.7
N2—C9—C10	100.10 (11)	C20—C19—H19	119.7
N2—C9—C16	114.01 (12)	C19—C20—C21	119.91 (15)
C10—C9—C16	113.99 (12)	C19—C20—H20	120
N2—C9—H9	109.5	C21—C20—H20	120
C10—C9—H9	109.5	O4—C21—C20	123.87 (14)
C16—C9—H9	109.5	O4—C21—C16	116.07 (13)
C15—C10—C11	122.13 (14)	C20—C21—C16	120.04 (14)
C15—C10—C9	111.54 (13)	O4—C22—H22A	109.5
C11—C10—C9	126.33 (14)	O4—C22—H22B	109.5
O3—C11—C10	122.89 (17)	H22A—C22—H22B	109.5
O3—C11—C12	122.94 (17)	O4—C22—H22C	109.5
C10—C11—C12	114.09 (16)	H22A—C22—H22C	109.5
C13—C12—C11	117.2 (2)	H22B—C22—H22C	109.5
C13—C12—H12A	108	C1—N1—C15	129.00 (12)
C11—C12—H12A	108	C1—N1—N2	123.30 (12)
C13—C12—H12B	108	C15—N1—N2	107.58 (11)
C11—C12—H12B	108	C8—N2—N1	124.21 (12)
H12A—C12—H12B	107.2	C8—N2—C9	125.25 (12)
C12—C13—C14	117.6 (2)	N1—N2—C9	110.53 (11)
C12—C13—H13A	107.9	C21—O4—C22	118.93 (14)
			
O1—C1—C2—C7	−178.79 (16)	N2—C9—C16—C17	−127.42 (14)
N1—C1—C2—C7	0.2 (2)	C10—C9—C16—C17	118.48 (15)
O1—C1—C2—C3	0.2 (2)	N2—C9—C16—C21	56.20 (18)
N1—C1—C2—C3	179.20 (14)	C10—C9—C16—C21	−57.90 (18)
C7—C2—C3—C4	−1.1 (2)	C21—C16—C17—C18	0.9 (2)
C1—C2—C3—C4	179.91 (15)	C9—C16—C17—C18	−175.63 (15)
C2—C3—C4—C5	0.3 (3)	C16—C17—C18—C19	−0.3 (3)
C3—C4—C5—C6	0.3 (3)	C17—C18—C19—C20	−0.5 (3)
C4—C5—C6—C7	−0.2 (3)	C18—C19—C20—C21	0.8 (3)
C3—C2—C7—C6	1.2 (2)	C19—C20—C21—O4	−178.87 (16)
C1—C2—C7—C6	−179.82 (14)	C19—C20—C21—C16	−0.2 (2)
C3—C2—C7—C8	−177.54 (14)	C17—C16—C21—O4	178.16 (14)
C1—C2—C7—C8	1.4 (2)	C9—C16—C21—O4	−5.4 (2)
C5—C6—C7—C2	−0.6 (2)	C17—C16—C21—C20	−0.6 (2)
C5—C6—C7—C8	178.20 (15)	C9—C16—C21—C20	175.82 (14)
C2—C7—C8—O2	177.83 (15)	O1—C1—N1—C15	1.5 (3)
C6—C7—C8—O2	−1.0 (2)	C2—C1—N1—C15	−177.53 (13)
C2—C7—C8—N2	−1.4 (2)	O1—C1—N1—N2	177.13 (15)
C6—C7—C8—N2	179.86 (14)	C2—C1—N1—N2	−1.9 (2)
N2—C9—C10—C15	−4.23 (16)	C10—C15—N1—C1	175.69 (15)
C16—C9—C10—C15	117.89 (14)	C14—C15—N1—C1	−5.3 (2)
N2—C9—C10—C11	175.59 (16)	C10—C15—N1—N2	−0.45 (16)
C16—C9—C10—C11	−62.3 (2)	C14—C15—N1—N2	178.51 (14)
C15—C10—C11—O3	178.88 (18)	O2—C8—N2—N1	−179.53 (14)
C9—C10—C11—O3	−0.9 (3)	C7—C8—N2—N1	−0.3 (2)
C15—C10—C11—C12	2.2 (3)	O2—C8—N2—C9	1.5 (2)
C9—C10—C11—C12	−177.6 (2)	C7—C8—N2—C9	−179.30 (13)
O3—C11—C12—C13	156.7 (3)	C1—N1—N2—C8	2.1 (2)
C10—C11—C12—C13	−26.6 (4)	C15—N1—N2—C8	178.49 (13)
C11—C12—C13—C14	45.2 (5)	C1—N1—N2—C9	−178.81 (13)
C12—C13—C14—C15	−35.9 (4)	C15—N1—N2—C9	−2.40 (15)
C11—C10—C15—N1	−176.72 (15)	C10—C9—N2—C8	−177.02 (14)
C9—C10—C15—N1	3.11 (17)	C16—C9—N2—C8	60.88 (19)
C11—C10—C15—C14	4.3 (3)	C10—C9—N2—N1	3.88 (14)
C9—C10—C15—C14	−175.83 (14)	C16—C9—N2—N1	−118.22 (13)
C13—C14—C15—C10	11.7 (3)	C20—C21—O4—C22	−20.5 (3)
C13—C14—C15—N1	−167.1 (2)	C16—C21—O4—C22	160.83 (16)

### Hydrogen-bond geometry (Å, º)

Cg3 is the centroid of ring C2-C7.

**Table:**

*D*—H···*A*	*D*—H	H···*A*	*D*···*A*	*D*—H···*A*
C22—H22*B*···O1^i^	0.96	2.43	3.379 (2)	168
C20—H20···*Cg*3^ii^	0.93	2.90	3.726 (2)	149

Symmetry codes: (i) −*x*+2, −*y*, −*z*+1; (ii) −*x*+2, *y*+1/2, −*z*+3/2.
