# Supplementary figures and images for: Crystal structure of 13-(2-meth­oxy­phenyl)-3,4-di­hydro-2H-indazolo[1,2-b]phthalazine-1,6,11(13H)-trione
Source: Acta Crystallogr E Crystallogr Commun. 2015 Jul 25;71(Pt 8):o604–5. doi: 10.1107/S2056989015013894 (PMC4571420; doi:10.1107/S2056989015013894)

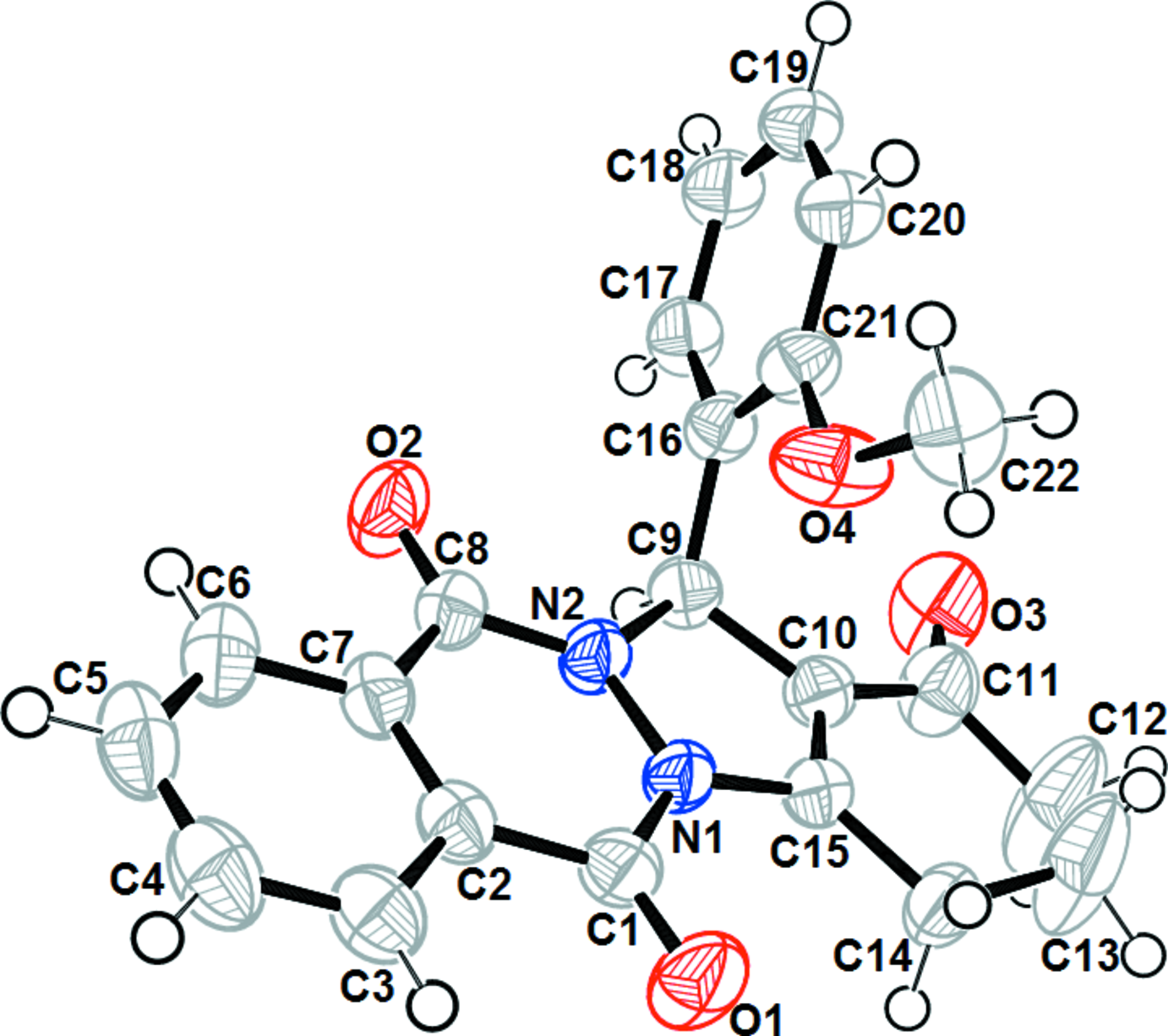

Supplement: Supplementary file 4 [file e-71-0o604-fig1.tif]

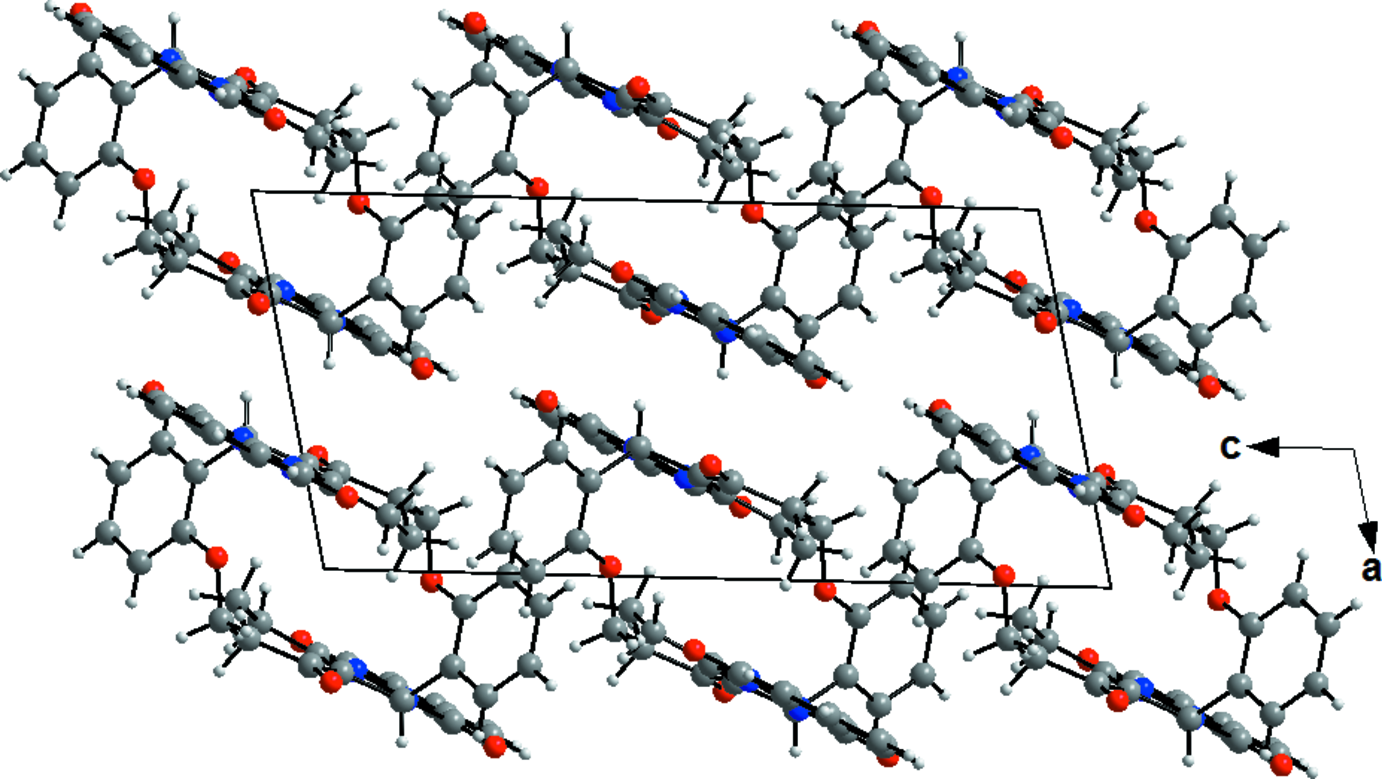

Supplement: Supplementary file 5 [file e-71-0o604-fig2.tif]
